# Repurposing atovaquone: Targeting mitochondrial complex III and OXPHOS to eradicate cancer stem cells

**DOI:** 10.18632/oncotarget.9122

**Published:** 2016-04-30

**Authors:** Marco Fiorillo, Rebecca Lamb, Herbert B. Tanowitz, Luciano Mutti, Marija Krstic-Demonacos, Anna Rita Cappello, Ubaldo E. Martinez-Outschoorn, Federica Sotgia, Michael P. Lisanti

**Affiliations:** ^1^ The Breast Cancer Now Research Unit, Institute of Cancer Sciences, Cancer Research UK Manchester Institute, University of Manchester, Manchester, UK; ^2^ The Manchester Centre for Cellular Metabolism (MCCM), Institute of Cancer Sciences, Cancer Research UK Manchester Institute, University of Manchester, Manchester, UK; ^3^ The Department of Pharmacy, Health and Nutritional Sciences, The University of Calabria, Cosenza, Italy; ^4^ Department of Medicine and Pathology, Albert Einstein College of Medicine, Bronx, NY, USA; ^5^ School of Environment and Life Sciences, University of Salford, Salford, UK; ^6^ The Sidney Kimmel Cancer Center, Philadelphia, PA, USA

**Keywords:** atovaquone, tumor-initiating cells (TICs), mitochondria, OXPHOS, cancer stem-like cells (CSCs)

## Abstract

Atovaquone is an FDA-approved anti-malarial drug, which first became clinically available in the year 2000. Currently, its main usage is for the treatment of pneumocystis pneumonia (PCP) and/or toxoplasmosis in immune-compromised patients. Atovaquone is a hydroxy-1,4-naphthoquinone analogue of ubiquinone, also known as Co-enzyme Q10 (CoQ10). It is a well-tolerated drug that does not cause myelo-suppression. Mechanistically, it is thought to act as a potent and selective OXPHOS inhibitor, by targeting the CoQ10-dependence of mitochondrial complex III. Here, we show for the first time that atovaquone also has anti-cancer activity, directed against Cancer Stem-like Cells (CSCs). More specifically, we demonstrate that atovaquone treatment of MCF7 breast cancer cells inhibits oxygen-consumption and metabolically induces aerobic glycolysis (the Warburg effect), as well as oxidative stress. Remarkably, atovaquone potently inhibits the propagation of MCF7-derived CSCs, with an IC-50 of 1 μM, as measured using the mammosphere assay. Atovaquone also maintains this selectivity and potency in mixed populations of CSCs and non-CSCs. Importantly, these results indicate that glycolysis itself is not sufficient to maintain the proliferation of CSCs, which is instead strictly dependent on mitochondrial function. In addition to targeting the proliferation of CSCs, atovaquone also induces apoptosis in both CD44+/CD24low/− CSC and ALDH+ CSC populations, during exposure to anchorage-independent conditions for 12 hours. However, it has no effect on oxygen consumption in normal human fibroblasts and, in this cellular context, behaves as an anti-inflammatory, consistent with the fact that it is well-tolerated in patients treated for infections. Future studies in xenograft models and human clinical trials may be warranted, as the IC-50 of atovaquone's action on CSCs (1 μM) is >50 times less than its average serum concentration in humans.

## INTRODUCTION

Cancer Stem-like Cells (CSCs) are a sub-population of cancer cells that have been shown to be resistant to conventional chemo- and radio-therapy [1–4]. Residual CSCs left behind after clinical treatment are considered responsible for the re-growth of tumors and for their metastatic dissemination [5, 6]. Thus, much effort has been devoted to the development of agents that are able to specifically target CSCs. Most approaches have focused on the targeting of signaling pathways necessary for their survival, expansion or self-renewal, as well as CSC-specific cell surface markers [7–10]. Other strategies include the possibility to induce CSC differentiation or the targeting of the CSC “niche” [11–14].

CSCs are strictly dependent on mitochondrial biogenesis, as well as mitochondrial function, for their clonal expansion. We have shown that pharmacological inhibition of the formation of new mitochondria is sufficient to block breast CSCs. In this regard, we have demonstrated that treatment with doxycycline, an inhibitor of mitochondrial ribosomes, or with XCT790, an inverse agonist of Estrogen-Related Receptor α (ERRα), a cofactor of PGC1α that is required for the transcription of mitochondrial genes and mitochondrial biogenesis [15, 16], was sufficient to reduce mammosphere formation and *bona-fide* CSC markers [17, 18]. Similarly, the antimicrobial tigecycline selectively killed acute myeloid leukemia stem cells, by inhibition of mitochondrial translation [19]. Moreover, treatment with oligomycin A, an inhibitor of the ATP synthase, greatly reduced mammosphere formation [20]. Similarly, metformin, which has complex I inhibitory effects, induced rapid apoptosis of pancreatic CSCs [21]. Salinomycin, an antibiotic that was recently identified as a selective inhibitor of CSCs [22] has been shown to reduce cell survival, at least partially, by impairing mitochondrial bioenergetic performance [23]. Finally, pyrvinium pamoate, an FDA-approved anti-parasitic agent, behaves as an OXPHOS inhibitor targeting mitochondrial complex II and efficiently prevents mammosphere formation in the nano-molar range, with an IC-50 of 50 nM [18]. However, pyrvinium pamoate is not absorbed efficiently from the gut, impeding its use for systemic anti-cancer therapy. Taken together, these studies provide a solid foundation and proof-of-concept for the new therapeutic strategy of targeting mitochondrial function to eradicate stem-like cancer cells.

In an ongoing search for targeted, yet safe, mitochondrial inhibitors, we identified atovaquone, a complex III inhibitor, that was originally developed to block the mitochondrial respiration of Plasmodium falciparum and other protozoa [24]. Atovaquone is a safe, FDA-approved drug, used for malaria prevention, and for the prevention and treatment of pneumocystis pneumonia and toxoplasmosis in HIV patients [25, 26]. Atovaquone can be administered alone as a liquid suspension (brand name Mepron) or in combination with Proguanil (brand name Malarone). Atovaquone is a highly lipophilic compound, with limited solubility in water. The bioavailability of atovaquone is dependent on its formulation and the diet, and its absorption is enhanced by high-fat food intake. Importantly, with current oral formulations, the average serum concentration of atovaquone in humans is > 50 μM.

Atovaquone is an extremely non-toxic OXPHOS inhibitor. Remarkably, attempts to suicide by overdosing on atovaquone, by taking three to 42-fold the normal dose, resulted in few, if any, side effects. Atovaquone [trans-2-[4-(4-chlorophenyl)cyclohexyl]-3-hydroxy-1,4- naphthalene-dione] is a quinone that functions as a competitive inhibitor of co-enzyme Q10, specifically inhibiting the mitochondrial electron transport chain in mitochondria isolated from Plasmodium falciparum at the cytochrome bc1 complex (Complex III) [27, 28]. Consistent with these findings, atovaquone has been shown to depolarize malarial mitochondria, resulting in a loss of mitochondrial function [24].

However, atovaquone efficacy was never previously tested in CSCs. Here, we set out to evaluate if atovaquone is an inhibitor of mitochondrial function in cancer cells and if it can be used as a targeted agent for breast CSCs.

## RESULTS

### Atovaquone, a ‘safe’ OXPHOS inhibitor that potently targets cancer stem cells

**Figure 1:**
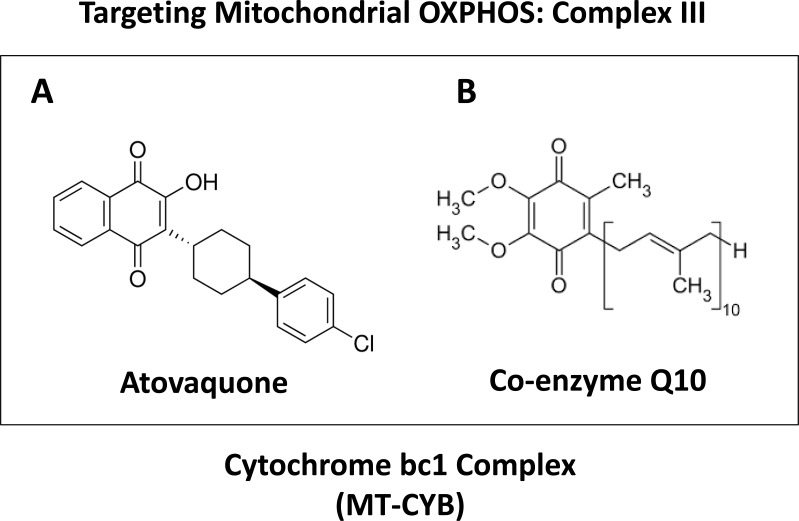
Atovaquone: Comparison with the structure of CoQ10 Note that atovaquone **A.** is highly structurally-related to CoQ10 **B.** and was first designed to target the MT-CYB subunit of Cytochrome bc1 (mitochondrial complex III) in malarial parasites.

The aim of this study is to evaluate if atovaquone (Figure 1) is a potent inhibitor of mitochondrial function in cancer cells and if it can be used as a targeted agent for breast CSCs.

**Figure 2:**
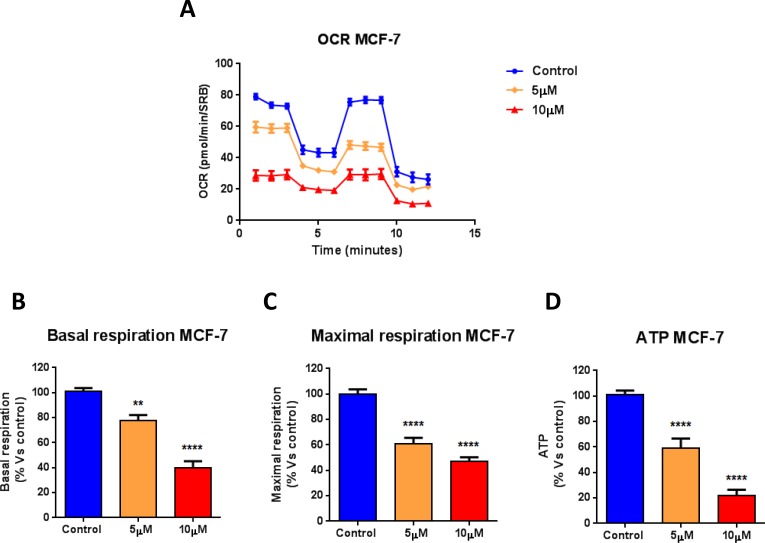
Atovaquone treatment inhibits the mitochondrial respiration of MCF7 breast cancer cells The metabolic profile of MCF7 cell monolayers treated with atovaquone (5μM and 10 μM) for 48 hours was assessed using the Seahorse XF-e96 analyzer. **A.** The tracing of 3 independent experiments is shown. **B.**, **C.**, **D.** Significant reductions in basal respiration, maximal respiration, and ATP levels were observed experimentally. **p < 0.001, ****p < 0.00001, one-way ANOVA and Student's *t*-test calculations.

**Figure 3:**
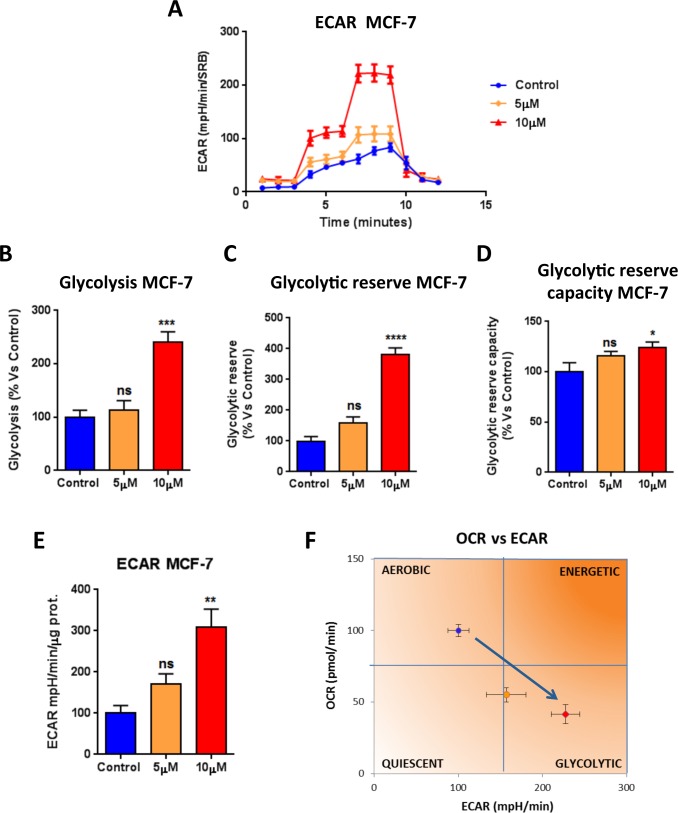
Atovaquone treatment increases glycolysis in MCF7 breast cancer cells The glycolytic profile of MCF7 cell monolayers treated with atovaquone (5μM and 10 μM) for 48 hours was assessed using the Seahorse XF-e96 analyzer. **A.** The tracing of 3 independent experiments is shown. **B.**, **C.**, **D.** Significant increases in glycolysis, glycolytic reserve, and glycolytic reserve capacity were observed experimentally. **E.** ECAR was also found increased. **F.** ECAR and OCR were plotted on the same graph. Note that atovaquone treatment (10 μM) shifts MCF7 cancer cells from an aerobic state to a glycolytic state. *p < 0.01, **p < 0.001, ***p < 0.0001, ****p < 0.00001, ns not significant, one-way ANOVA and Student's *t*-test calculations.

Towards this end, the metabolic profile of MCF7 breast cancer cells treated with increasing concentrations of atovaquone was assessed using the Seahorse XF-e96 analyzer. Atovaquone treatment markedly inhibits the mitochondrial respiration of MCF7 cells, with significant reductions in basal respiration, maximal respiration, and ATP levels (Figure 2). Moreover, atovaquone treatment increases aerobic glycolysis in MCF7 cells, with significant increases in glycolysis, glycolytic reserve, and glycolytic reserve capacity (Figure 3A-3E). The calculation of the Cell Energy Profile, obtained by plotting Oxygen Consumption Rates (OCR) against Extra-Cellular Acidification Rates (ECAR), demonstrates that atovaquone shifts MCF7 cells from an oxidative state to a glycolytic state (Figure 3F).

**Figure 4:**
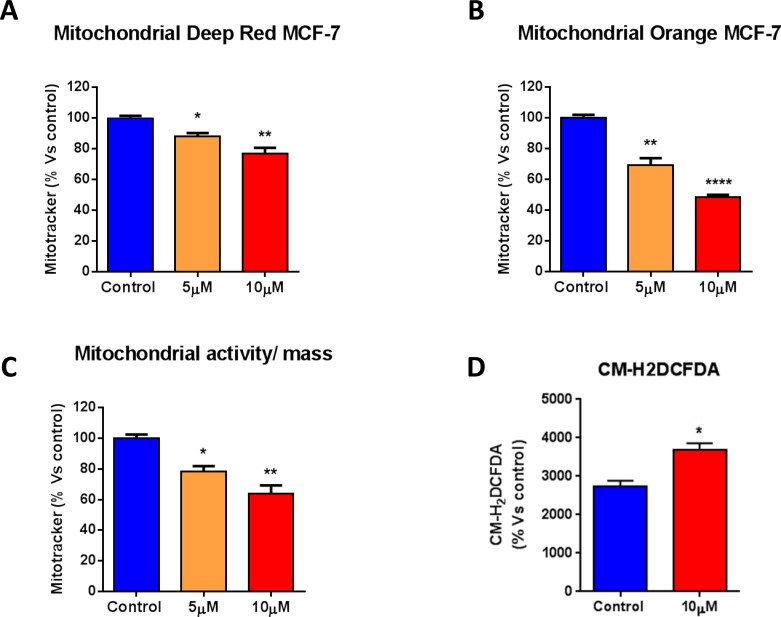
Atovaquone treatment decreases mitochondrial mass and membrane potential, with an increase in ROS levels MCF7 cells were treated with atovaquone (5μM or 10 μM) for 48 hours, and stained with various metabolic probes and analyzed by FACS. **A.**, **B.** Atovaquone decreases mitochondrial mass, as assessed with MitoTracker Deep-Red, and mitochondrial membrane potential, as assessed with MitoTracker Orange. **C.** The ratio of mitochondrial membrane potential (MitoTracker Orange) *versus* mitochondrial mass (MitoTracker Deep-Red) was calculated, showing that atovaquone significantly decreases mitochondrial membrane potential per mitochondria. **D.** ROS levels were determined using the CM-H_2_DCFDA probe by FACS. Note that atovaquone (10 μM) increases ROS levels, as expected. *p < 0.01, **p < 0.001,, ****p < 0.00001, one-way ANOVA and Student's *t*-test calculations.

To further characterize the mitochondrial function of MCF7 cells after treatment with atovaquone, MCF7 cells were stained with various metabolic probes and analyzed by FACS. Atovaquone decreases mitochondrial mass, as assessed with MitoTracker Deep-Red (Figure 4A), and mitochondrial membrane potential, as assessed with MitoTracker Orange (Figure 4B). The ratio of mitochondrial membrane potential (MitoTracker Orange) *versus* mitochondrial mass (MitoTracker Deep-Red) was calculated, showing that atovaquone significantly decreases the mitochondrial membrane potential per mitochondria (Figure 4C). We next determined Reactive Oxygen Species (ROS) levels using the CM-H_2_DCFDA probe by FACS. Note that atovaquone increases ROS levels, as expected (Figure 4D). Taken together, these data are consistent with the fact that atovaquone acts as a mitochondrial inhibitor in cancer cells, greatly impairing mitochondrial respiration, with a compensatory increase in glycolysis.

**Figure 5:**
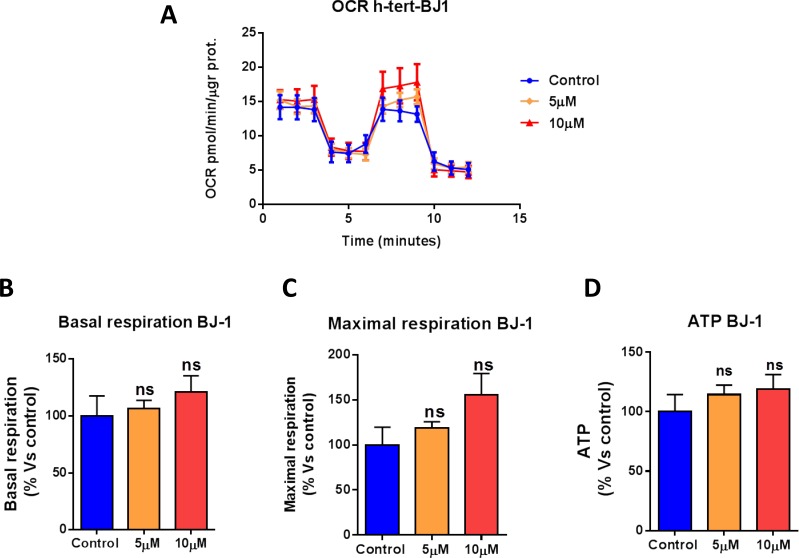
Atovaquone treatment does not affect the mitochondrial respiration of normal human fibroblasts The metabolic profile of normal human fibroblast (hTERT-BJ1) monolayers treated with atovaquone (5μM and 10 μM) for 48 hours was assessed using the Seahorse XF-e96 analyzer. **A.** The tracing of 3 independent experiments is shown. **B.**, **C.**, **D.** Note that atovaquone does not significantly reduce basal respiration, maximal respiration, or ATP levels of normal human fibroblasts. ns, not significant; one-way ANOVA and Student's *t*-test calculations.

**Figure 6:**
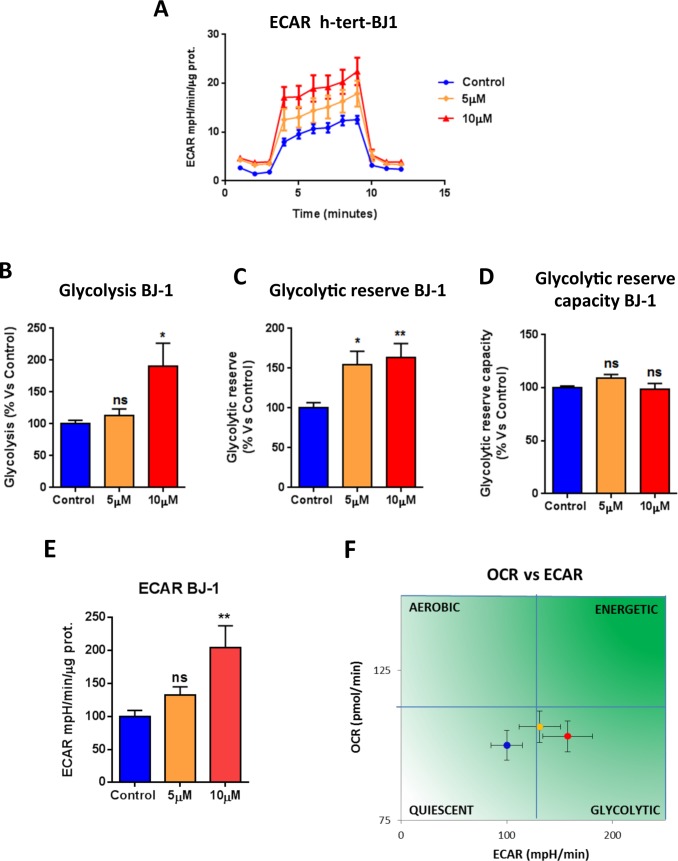
Atovaquone treatment increases glycolysis in normal human fibroblasts The glycolytic profile of normal human fibroblast (hTERT-BJ1) monolayers treated with atovaquone (5μM and 10 μM) for 48 hours was assessed using the Seahorse XF96 analyzer. **A.** The tracing of 3 independent experiments is shown. **B.**, **C.**, **D.** Significant increases in glycolysis, and glycolytic reserve were observed experimentally. **E.** ECAR was also found increased. **F.** ECAR and OCR were plotted on the same graph. Note that atovaquone treatment does not change the metabolic state of normal human fibroblasts. *p < 0.01, **p < 0.001, ***p < 0.0001, ****p < 0.00001, ns not significant, one-way ANOVA and Student's *t*-test calculations.

We next asked if atovaquone functions as a mitochondrial inhibitor specifically for cancer cells, or if it affects all types of mammalian cells. To this end, we assessed the metabolic profile of normal human fibroblasts treated with atovaquone for 48 hours using the Seahorse XF-e96 analyzer. Surprisingly, atovaquone does not inhibit at all the basal respiration, maximal respiration, and ATP levels of normal human fibroblasts, but rather we observed a slight, but not significant, increase in mitochondrial respiration (Figure 5A-5D). The glycolytic profile of normal human fibroblasts treated with atovaquone was also assessed using the Seahorse XF-e96 analyzer. Significant increases in glycolysis, and glycolytic reserve were observed experimentally, indicating that atovaquone increases glycolysis in normal human fibroblasts (Figure 6A-6E). Interestingly, in contrast to what we observed for MCF7 cancer cells, plotting of OCR against ECAR shows that atovaquone does not alter the metabolic state of normal human fibroblasts (Figure 6F).

**Figure 7:**
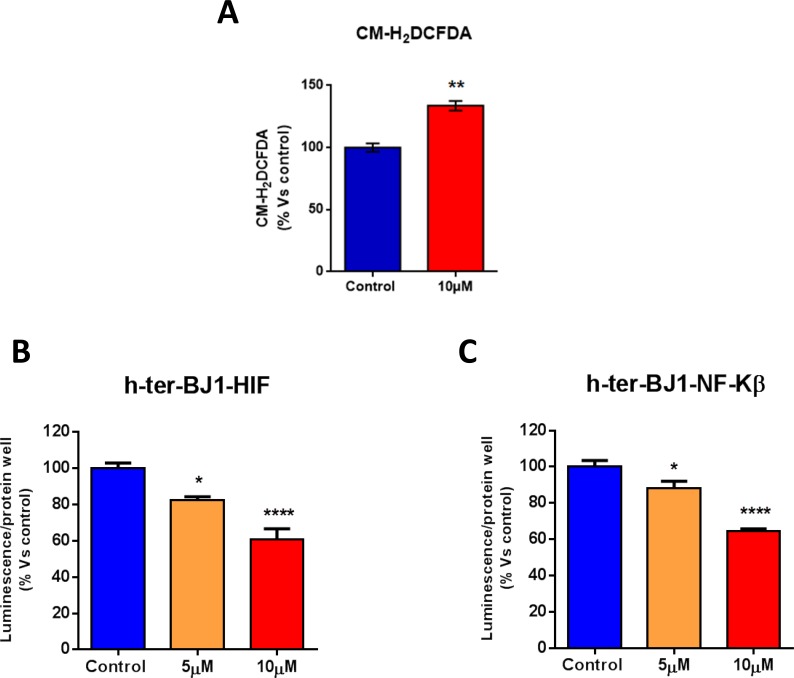
Atovaquone treatment does not induce pro-inflammatory or stress-induced responses in human normal fibroblasts **A.** ROS levels were measured in normal human fibroblasts treated with atovaquone (10 μM) or DMSO for 48 hours. Note that atovaquone increases ROS levels. **B.**, **C.** The activation of HIF and NFκB pathways was monitored using Luc-reporter fibroblasts carrying HIF- and NFκB-Luc reporter elements. Luciferase assays were performed on cells treated with atovaquone (5μM and 10 μM) or DMSO control for 48 hours. Luminescence was normalized using SRB (total proteins), as a measure of cell viability. Note that atovaquone treatment decreases the activation of HIF (B) and NFκB (C) pathways in normal fibroblasts, in a dose-dependent manner. *p < 0.01, **p < 0.001, ****p < 0.00001 evaluated with one-way ANOVA.

We next measured ROS levels in human fibroblasts treated with atovaquone. Note that atoquavone increases ROS levels (Figure 7A). An increase in ROS levels may induce the activation of stress-induced responses pathways, such as HIF pathway, and/or pro-inflammatory signaling, such as NFκB, which are potentially toxic for cells. To monitor the activation of HIF and NFκB pathways after atovaquone treatment, we employed reporter fibroblasts carrying HIF- and NFκB-Luc reporter elements. Vehicle alone (DMSO) control cells were run in parallel. Surprisingly, atovaquone treatment decreases the activation of HIF and NFκB pathways in normal fibroblasts, in a dose-dependent manner (Figure 7B-7C). These results indicate that atovaquone inhibits inflammatory or stress-induced responses, consistent with its safe use in the clinic.

**Figure 8:**
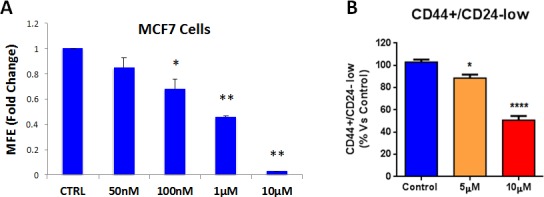

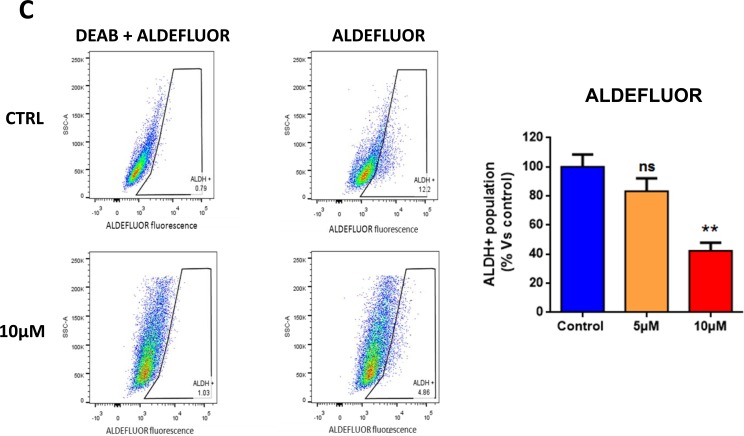
Atovaquone treatment inhibits MCF7-derived CSCs **A.** MCF7 cells were plated for mammosphere assays in the presence of increasing concentrations of atovaquone or vehicle alone. Atovaquone dose-dependently inhibits MCF7 mammosphere formation efficiency (MFE) at 5 days, with an IC-50 of ~1 μM. *p < 0.05, **p < 0.00001, evaluated by Student's t test. **B.** MCF7 cells were pre-treated with atovaquone (5μM and 10 μM) as monolayers for 48 hours and then re-plated on low-attachment plates in the absence of atovaquone, for anoikis assay for 12 hours. Expression of CSC markers (CD24 and CD44) was analyzed by FACS. Note that atovaquone pre-treatment dose-dependently decreases the CD44+/CD24-^low^ cell population, which is considered the CSC population. **C.** MCF7 cells were pre-treated with atovaquone (5μM and 10 μM) as monolayers for 48 hours and then assessed for ALDEFLUOR activity, an independent marker of CSCs. Each sample was normalized using diethylaminobenzaldehyde (DEAB), a specific ALDH inhibitor, as negative control. C. The tracing of representative samples is shown. Note that atovaquone treatment decreases the ALDH-positive cell population by 50%. *p < 0.01, ****p < 0.00001 evaluated with one-way ANOVA.

Having firmly established that atovaquone specifically inhibits mitochondrial respiration in MCF7 cancer cells, but not in normal fibroblasts, we then asked if atovaquone could halt the propagation of the CSC population. To this end, MCF7 cells were plated for mammosphere assays in the presence of increasing concentrations of atovaquone or vehicle alone for 5 days. Atovaquone dose-dependently inhibits MCF7 mammosphere formation, with an IC-50 of ~1 μM (Figure 8A). To independently validate these results, the expression of CSC markers (CD24 and CD44) was analyzed by FACS. Notably, atovaquone pre-treatment dose-dependently decreases the CD44+/CD24-^low^ cell population, which is considered to be the CSC population (Figure 8B). Next, we assessed ALDEFLUOR activity, an independent marker of CSCs, using MCF7 cells pre-treated with atovaquone. A specific ALDH inhibitor (diethylaminobenzaldehyde - DEAB) was used for each sample, as a negative control. Note that atovaquone treatment significantly decreases the ALDH-positive cell population by 50% (Figure 8C). Thus, these results indicate that atovaquone treatment also induces apoptosis in MCF7-derived CSCs, during exposure to anchorage-independent conditions (i.e., anoikis), for 12 hours.

**Figure 9:**
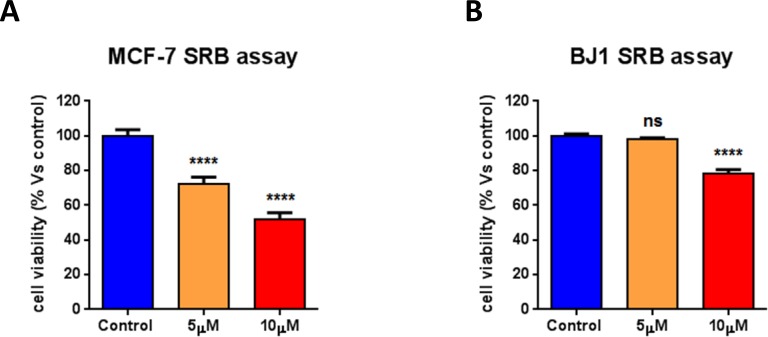
Atovaquone treatment preferentially inhibits CSCs, with minor effects on the viability of normal human fibroblasts MCF7 cells and normal human fibroblasts (hTERT-BJ1) were treated with atovaquone (5μM and 10 μM) for 5 days. Cell viability was assessed by SRB assay. **A.** Atovaquone treatment inhibits MCF7 cell viability with an IC-50 of ~10 μM, indicating that atovaquone preferentially inhibits CSCs (compare with Figure 8A), relative to the ‘bulk’ cancer cells. **B.** Atovaquone treatment slightly inhibits normal human fibroblast viability, consistent with its safe use in humans. ****p < 0.00001, ns not significant, evaluated with one-way ANOVA.

We next asked if atovaquone action is specific for CSCs, or if it affects the viability of the total cancer cell population or of normal human fibroblasts. To this end, cell viability was assessed on MCF7 cells and normal human fibroblasts (hTERT-BJ1) treated with atovaquone for 5 days. Interestingly, atovaquone treatment inhibits MCF7 cell viability with an IC-50 of ~10 μM (Figure 9A), indicating that atovaquone preferentially inhibits CSCs (compare with Figure 8A), relative to the ‘bulk’ cancer cells. Thus, atovaquone is about 10 times more potent in the inhibition of CSCs propagation (IC-50 of ~1 μM). Moreover, atovaquone treatment only slightly inhibits normal human fibroblast viability, consistent with its safe use in humans (Figure 9B).

**Figure 10:**
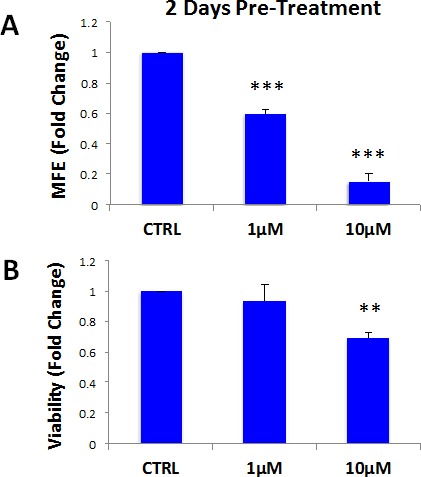
Atovaquone pre-treatment preferentially inhibits CSCs, with minor effects on the viability of bulk cancer cells MCF7 cells were pre-treated with atovaquone (1μM and 10 μM) as monolayers for 2 days. Then, cells re-plated on low-attachment plates in the absence of atovaquone, for mammosphere assay for 5 days, or directly assessed for cell growth and viability. **A.** Atovaquone pre-treatment inhibits MCF7 cell mammosphere formation, in a dose-dependent fashion (~85% inhibition with 10 μM atovaquone). ***p < 0.0001 evaluated by Student's t test **B.** Under the same conditions, atovaquone has mild effects on the viability of bulk cancer cells (~30% inhibition of viability with 10 μM atovaquone). **p < 0.001 evaluated by Student's t test. MFE: mammosphere forming efficiency.

Finally, we asked if atovaquone could still target CSCs in a heterogeneous attached cell population, when they are mixed with the bulk of the cancer cells. To this end, MCF7 cells were pre-treated with atovaquone as monolayers for 2 days, and then cells were collected and re-plated on low-attachment plates in the absence of atovaquone, for mammosphere assays, or directly assessed for cell viability. Interestingly, atovaquone pre-treatment preferentially inhibits CSCs, in a dose-dependent fashion (~85% inhibition with 10 μM atovaquone) (Figure 10A), with more moderate effects on the viability of bulk cancer cells (only ~30% inhibition with 10 μM atovaquone) (Figure 10B). These results indicate that atovaquone is able to target CSCs also when they are in a heterogeneous attached cell population (IC-50 ~ 1 μM), when they are mixed with bulk cancer cells.

### Validation of the clinical relevance of mitochondrial complex III in human breast cancer patients, in both ER(+) and ER(−) epithelial sub-types

**Figure 11:**
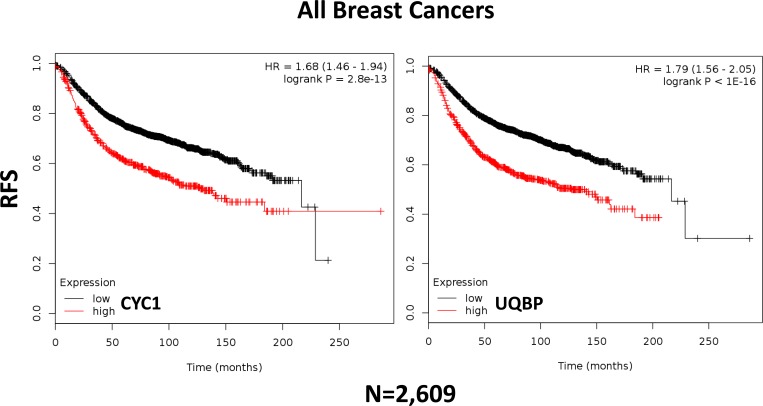
Kaplan-Meier (K-M) analysis of the prognostic value of two components of mitochondrial complex III Results of recurrence-free survival analysis (RFS) are shown, over nearly a 20-year period of follow-up, for two mitochondrial markers for all breast cancers taken as a single group (*N* = 2,609). Note that higher levels of these two mitochondrial markers are significantly associated with tumor recurrence. See the *Methods section* for further details of how the analysis was carried out. Similar results were obtained in other sub-types of breast cancer, including ER(+) and ER(−), and are included as Supplementary Figures S1 and S2.

In order to assess the possible clinical relevance of mitochondrial complex III, we determined if different components of the complex show any prognostic value in human breast cancer patient cohorts, with long-term follow-up (approaching 20 years). These results are summarized in Tables 1 and 2. Corresponding Kaplan-Meier (K-M) analysis curves are included in Figure 11, and as Supplementary Figures S1 and S2 (Panels A-I).

**Table 1:** High CYC1 mRNA Levels are Associated with Increased Tumor Recurrence, in Both ER(+) and ER(−) Breast Cancer Patients

Cancer Subtype	HR	Log-Rank	N
All Subtypes	1.68	2.8e-13	2,609
ER(−)	1.53	0.002	584
ER(−)/Basal	1.69	0.0008	436
ER(+)	1.65	1.1e-09	2,025
ER(+)/Endocrine Therapy	1.73	0.0007	698
ER(+)/Endocrine Therapy Luminal A/LN(+)	1.96	0.02	145
ER(+)/Endocrine Therapy Luminal A/LN(−)	2.94	0.0098	286
ER(+)/Endocrine Therapy Luminal B	1.97	0.002	247
ER(+)/Endocrine Therapy Luminal B/LN(−)	3.11	0.001	117

CYC1, Cytochrome C1; ER, estrogen receptor; LN, lymph node; HR, hazard ratio; N, the number of patients in a given subtype or group. The vast majority of patients receiving endocrine therapy were treated with tamoxifen.

**Table 2:** High UQBP mRNA Levels are Associated with Increased Tumor Recurrence, in Both ER(+) and ER(−) Breast Cancer Patients

Cancer Subtype	HR	Log-Rank	N
All Subtypes	1.79	<1e-16	2,609
ER(−)	1.86	3.6e-06	584
ER(−)/Basal	1.91	3.3e-05	436
ER(+)	1.75	7.4e-13	2,025
ER(+)/Endocrine Therapy	2.22	8.5e-08	698
ER(+)/Endocrine Therapy Luminal A/LN(+)	3.42	1.9e-05	145
ER(+)/Endocrine Therapy Luminal A/LN(−)	2.04	0.016	286
ER(+)/Endocrine Therapy Luminal B	2.28	0.0001	247
ER(+)/Endocrine Therapy Luminal B/LN(−)	4.00	4.1e-05	117

UQBP, Ubiquinol-Cytochrome C Reductase Binding Protein; ER, estrogen Receptor; LN, lymph node; HR, hazard ratio; N, the number of patients in a given subtype or group. The vast majority of patients receiving endocrine therapy were treated with tamoxifen.

More specifically, high mRNA expression levels of CYC1 (Cytochrome C1) and UQBP (Ubiquinol-Cytochrome C Reductase Binding Protein), key components of complex III, are both associated with significantly reduced progression-free survival (i.e., higher tumor recurrence). Similar results were obtained in both ER(+) and ER(−) patients, as well as in ER(+) sub-types (luminal A and luminal B patients). Note that high levels of CYC1 and UQBP were also associated with progression in patients that received endocrine therapy, which is indicative of a clinical association with endocrine therapy-resistance.

Thus, in the future, elevated levels of expression of CYC1 and UQBP could be used to identify high-risk ER(+) and ER(−) breast cancer patients that might benefit from treatment with atovaquone, a complex III inhibitor. In this context, CYC1 and UQBP could also be used as companion diagnostics, to guide the use of mitochondrially-targeted atovaquone therapy.

## DISCUSSION

**Figure 12:**
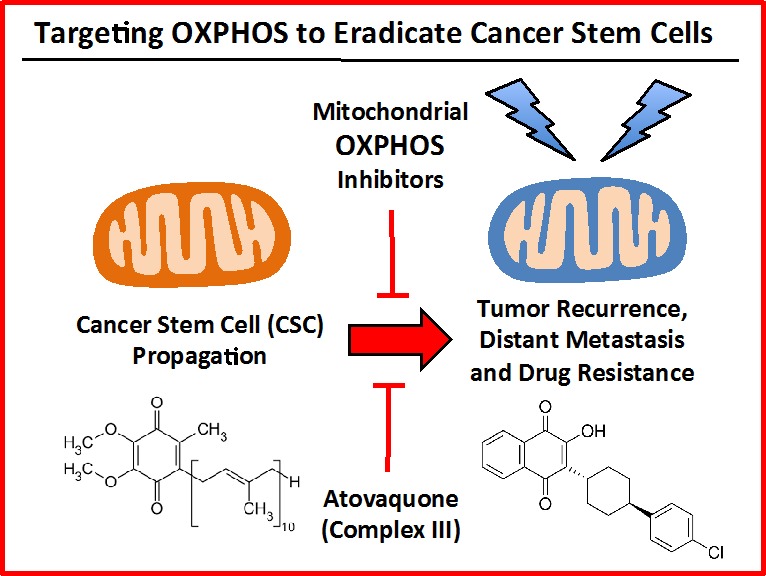
Targeting OXPHOS to eradicate CSCs: Repurposing atovaquone The propagation of CSCs is thought to underpin the molecular and cellular basis of tumor recurrence, distant metastasis and resistance to chemo- and radio-therapy. Here, we suggest the use of a “safe” mitochondrial OXPHOS inhibitor to eradicate CSCs, which are addicted to mitochondrial function. More specifically, atovaquone (shown at right) inhibits mitochondrial complex III, by effectively competing with the binding of CoQ10 (shown at left).

Atovaquone is an FDA-approved anti-parasitic drug that was first developed to target chloroquine-resistant malaria [25]. Currently, its major use is for managing pneumocystis pneumonia (PCP) and toxoplasmosis infections in HIV-positive patients [26]. Structurally, atovaquone resembles CoQ10 and competitively inhibits its binding to the Cytochrome b subunit (MT-CYB) of mitochondrial complex III [27, 28]. As a consequence, here we explored the possibility that atovaquone could be repurposed for targeting mitochondrial complex III in breast cancer cells (summarized in Figure 12). To test this hypothesis, we first examined its metabolic effects on MCF7 cells, using the Seahorse XF-e96, to measure mitochondrial function and glycolysis. Our results directly show that atovaquone significantly inhibits oxygen consumption and ATP production in the low micromolar range. Moreover, atovaquone treatment significantly induced aerobic glycolysis (a.k.a., the Warburg effect). In this context, we demonstrated that atovaquone reduces both mitochondrial mass and membrane potential, but increased overall ROS production. Importantly, atovaquone potently inhibited the propagation of CSCs, with an IC-50 of 1 μM, as measured using the mammosphere assay. Interestingly, this concentration is > 60 times lower than the average serum concentration achieved in patients. Remarkably, atovaquone treatment had little or no effect on normal human fibroblasts.

When atovaquone suspension was administered in humans with food at the standard regimen of 750 mg twice daily, the average steady-state plasma concentration was 21.0 ± 4.9 μg/mL, and the minimum plasma concentration was 16 ± 3.8 μg/mL [29]. It should be noted that in our experiments we have effectively ablated mammosphere formation with 10 μM atovaquone, which corresponds to 3.66 μg/mL. Thus, the clinically relevant, therapeutic plasma concentration of atovaquone is 5-times higher than the concentration that completely blocks the expansion of CSCs.

A thorough search of the literature revealed only one previous paper investigating the effects of atovaquone on cancer cell lines. Although mainly focused on the cytotoxicity and apoptosis-inducing activity of atovaquone derivatives, this report shows that atovaquone effectively inhibited the growth of Du145 prostate cancer cells, with an IC-50 of approximately 30 μM [30]. So, our current results are consistent with these overall findings. However, these authors did not evaluate the effects of atovaquone on CSCs.

Interestingly, it is well known that atovaquone binds directly to the Cytochrome b component of the Cytochrome bc1 complex, in the mitochondria of malarial parasites. This has been further verified by a mutational analysis and by determining the crystal structure of atovaquone bound to the Cytochrome b molecule, also known as MT-CYB [31, 32]. However, very little is known about the role of MT-CYB and the Cytochrome bc1 complex in cancer.

Genetic evidence of a role for MT-CYB in cancer comes from studies on breast and colon cancers. A recent study used targeted sequencing of mitochondrial DNA in high-risk breast cancer patients negative for BRCA1/2 mutations to provide new clinical evidence for MT-CYB variants as a risk factor for hereditary breast cancers [33]. Interestingly, they observed that two mitochondrial genes, namely MT-ATP6 (complex V) and MT-CYB (complex III) showed the highest number of variants in the high-risk breast cancer patients. In further support of these findings, 4 homoplasmic and 6 heteroplasmic alterations were found in the mitochondrial DNA of human colon cancer cell lines; among these changes, a homoplasmic mutation in MT-CYB (G14804A) results in amino acid substitutions in highly conserved residues [34]. Two homoplasmic mutations in MT-CYB [G14985A (R80H) and T15572C (F276L)] were independently observed in another cohort of human colon cancer patients [35]. The fact that these mutations are homoplasmic indicates that they provide both a strong replicative and proliferative advantage. Interestingly, the two latter mutations in MT-CYB did not result in any observable defects in OXPHOS, indicating that the mitochondria bearing these mutations remained highly functional [35].

MT-CYB mutations have also been detected in human bladder carcinomas [36, 37]. For example, a 21-bp in-frame deletion results in a loss of 7-amino acids in the MT-CYB protein product. Cells over-expressing this “activated mutant” of MT-CYB showed significant increases in cell proliferation and tumor growth, due to NFkB hyper-activation, increased oxygen consumption rates and resistance to apoptosis. This activated mutant of MT-CYB, specifically associated with bladder cancer, also functionally increased anchorage-independent growth and mitochondrial biogenesis, as well as mt-DNA content [36, 37].

The Cytochrome bc1 complex contains two major subunits: Cytochrome b (MT-CYB) and Cytochrome c1 (CYC1). Very little is known about the role of CYC1 in cancer pathogenesis. However, silencing of CYC1 (using an shRNA approach) in osteosarcoma cell lines effectively inhibits cellular proliferation, sensitizes the cells to apoptotic cell death and inhibits their ability to form tumors in xenograft models [38]. In their model system, the authors also demonstrated that CYC1 silencing significantly reduced mitochondrial complex III activity.

Interestingly, several reports indicate that the expression levels of mitochondrial complex III subunits are altered in human breast cancers [39, 40]. For example, ubiquinol cytochrome c reductase (UQCRFS1) is amplified in a subset of breast cancers and is associated with higher tumor grade [40]. In addition, there appears to be a relationship between tamoxifen-sensitivity and complex III. More specifically, inhibition of mitochondrial complex III enhances the cytotoxicity of tamoxifen in MCF7 cells [41]. This synergy may stem from the observation that tamoxifen may act directly on mitochondria, as an inhibitor of complex III function [42].

Taken together with our current data, these results suggest that atovaquone treatment may be an effective new strategy for targeting the Cytochrome bc1 complex (especially MT-CYB and CYC1) in cancer, to prevent the proliferative expansion of CSCs and tumor growth. Further pre-clinical studies to test atovaquone efficacy are clearly warranted. In direct support of the idea that targeting mitochondrial complex III is a promising therapeutic strategy, Kwon, Jung and colleagues have developed a series of small molecules that successfully inhibited the activity of complex III (UQBP; ubiquinol-cytochrome c reductase binding protein) [43, 44]. Interestingly, these small molecules also dramatically reduced tumor growth in pre-clinical xenograft models (harboring U87MG glioblastoma cells), without showing any *in vitro* and *in vivo* toxicity. However, these novel molecules will need to undergo phase I clinical trials.

In contrast, atovaquone is already FDA-approved and can immediately enter phase II clinical trails, potentially saving 10 years in development time and millions of dollars. Thus, the repurposing of atovaquone may be a more effective translational approach, to more rapidly bring mitochondrial complex III inhibitors to the clinic.

## MATERIALS AND METHODS

### Materials

MCF7 breast cancer cell lines were purchased from the ATCC. Human immortalized fibroblasts (hTERT-BJ1) were purchased from Clontech, Inc. Cells were cultured in Dulbecco's modified Eagle's medium (DMEM), supplemented with 10% FBS (fetal bovine serum), 2 mM GlutaMAX, and 1% Pen-Strep in a 37°C humidified atmosphere containing 5% CO_2_, unless otherwise noted. Gibco-brand cell culture media (DMEM/F12) was purchased from Life Technologies. Atovaquone was purchased from Sigma-Aldrich.

### Seahorse XFe-96 metabolic flux analysis

Extracellular acidification rates (ECAR) and real-time oxygen consumption rates (OCR) for MCF7 cells and fibroblasts treated with atovaquone were determined using the Seahorse Extracellular Flux (XFe-96) analyzer (Seahorse Bioscience, MA, USA) [17]. 10,000 cells per well were seeded into XFe-96 well cell culture plates, and incubated overnight to allow attachment. Then, cells were treated with atovaquone (5μM and 10 μM) for 48 hours. Vehicle alone (DMSO) control cells were processed in parallel. After 48 hours of incubation, cells were washed in pre-warmed XF assay media (or for OCR measurement, XF assay media supplemented with 10mM glucose, 1mM Pyruvate, 2mM L-glutamine and adjusted at 7.4 pH). Cells were then maintained in 175 μL/well of XF assay media at 37°C, in a non-CO_2_ incubator for 1 hour. During the incubation time, we loaded 25 μL of 80mM glucose, 9μM oligomycin, and 1M 2-deoxyglucose (for ECAR measurement) or 10μM oligomycin, 9μM FCCP, 10μM rotenone, 10μM antimycin A (for OCR measurement), in XF assay media into the injection ports in the XFe-96 sensor cartridge. Measurements were normalized by protein content (SRB or Bradford assay). Data set was analyzed by XFe-96 software and GraphPad Prism software, using one-way ANOVA and Student's *t*-test calculations. All experiments were performed three times independently.

### Mitochondrial staining

Mitochondrial activity was assessed with MitoTracker Orange (#M7510, Invitrogen), whose accumulation in mitochondria is dependent upon membrane potential. Mitochondrial mass was determined using MitoTracker Deep-Red (#M22426, Invitrogen), localizing to mitochondria regardless of mitochondrial membrane potential. MCF7 cells were treated with atovaquone (5 μM and 10 μM) for 48 hours. Vehicle alone (DMSO) control cells were processed in parallel. After 48 hours, cells were incubated with pre-warmed MitoTracker staining solution (diluted in PBS/CM to a final concentration of 10 nM) for 30-60 minutes at 37°C. All subsequent steps were performed in the dark. Cells were washed in PBS, harvested, and re-suspended in 300 μL of PBS/CM. Cells were then analyzed by flow cytometry using Fortessa (BD Bioscience). Data analysis was performed using FlowJo software.

### ROS staining

Reactive oxygen species (ROS) production was measured using CM-H_2_DCFDA (C6827, Invitrogen), a cell-permeable probe that is non-fluorescent until oxidation within the cell. Cells were treated with 10 μM atovaquone for 48 hours. Vehicle alone (DMSO) control cells were processed in parallel. After 48 hours, cells were washed with PBS, and incubated with CM-H_2_DCFDA (diluted in PBS/CM to a final concentration of 1 μM) for 20 minutes at 37°C. All subsequent steps were performed in the dark. Cells were rinsed, harvested, and re-suspended in PBS/CM. Cells were then analyzed by flow cytometry using the Fortessa (BD Bioscience). ROS levels were estimated by using the mean fluorescent intensity of the viable cell population. The results were analyzed using FlowJo software (Tree star Inc.).

### Mammosphere culture

A single cell suspension was prepared using enzymatic (1x Trypsin-EDTA, Sigma Aldrich, #T3924), and manual disaggregation (25 gauge needle) [45]. Cells were plated at a density of 500 cells/cm^2^ in mammosphere medium (DMEM-F12/B27/20ng/ml EGF/PenStrep) in non-adherent conditions, in culture dishes coated with (2-hydroxyethylmethacrylate) (poly-HEMA, Sigma, #P3932). Then, cells were treated with increasing concentrations of atovaquone (range 50 nM to 10 μM). Vehicle alone (DMSO) control cells were processed in parallel. Cells were grown for 5 days and maintained in a humidified incubator at 37°C. After 5 days for culture, spheres > 50 μm were counted using an eye piece graticule, and the percentage of cells plated which formed spheres was calculated and is referred to as percentage mammosphere formation, and was normalized to one (1 = 100 %MFE). Similar results were also obtained when cells were seeded at a density of 200 cells/cm^2^.

### CD44/CD24 analysis

1 × 10^5^ MCF7 cells were treated with atovaquone (5 μM and 10 μM) for 48 hours in 6-well plates, grown as a monolayer. Then, cells were trypsinized and seeded in low-attachment plates in mammosphere media. After 12 hours, MCF7 cells were spun down and incubated with CD24 (IOTest CD24-PE, Beckman Coulter) and CD44 (APC mouse Anti-Human CD44, BD Pharmingen cat.559942) antibodies for 15 minutes on ice. Cells were rinsed twice and incubated with LIVE/DEAD dye (Fixable Dead Violet reactive dye; Invitrogen) for 10 minutes. Samples were then analyzed by FACS (Fortessa, BD Bioscence). Only the live population, as identified by the LIVE/DEAD dye staining, was analyzed for CD24/CD44 expression. Data were analyzed using FlowJo software.

### ALDEFLUOR assay and separation of the ALDH-positive population by FACS

ALDH activity was assessed in MCF7 cells after treatment with atovaquone (5μM and 10μM) or vehicle control. The ALDEFLUOR kit (StemCell Technologies, Vancouver, Canada) was used to isolate the population with high ALDH enzymatic activity by FACS (Fortessa, BD Bioscence). Briefly, 1 × 10^5^ MCF7 cells were incubated in 1ml ALDEFLUOR assay buffer containing ALDH substrate (5μl/ml) for 40 minutes at 37°C. In each experiment, a sample of cells was stained under identical conditions with 30 μM of diethylaminobenzaldehyde (DEAB), a specific ALDH inhibitor, as negative control. The ALDEFLUOR-positive population was established in according to the manufacturer's instructions and was evaluated in 2 × 10^4^ cells.

### Pre-treatment of monolayers with atovaquone

After treatment with atovaquone in monolayers (1 μM or 10 μM for 2 days), MCF7 cells were trypsinized and seeded for mammosphere cultures. In parallel, cells were assessed for cell viability or cell growth. Cell growth was quantitatively measured using an Automated Cell Counter (Biorad), and cell viability was assessed by labeling with Trypan blue (Sigma).

### SRB assay

Cell viability was assessed by sulphorhodamine (SRB) assay, based on the measurement of cellular protein content. After treatment with atovaquone for 5 days in 96 well plates, cells were fixed with 10% trichloroacetic acid (TCA) for 1 hour in cold room, and dried overnight at room temperature. Then, cells were incubated with SRB for 15 min, washed twice with 1% acetic acid, and air dried for at least 3 hour. Finally, the protein-bound dye was dissolved in 10 mM Tris pH 8.8 solution and read using a plate reader at 540 nm.

### Evaluation of HIF and NFκB signaling pathways

The Cignal Lenti reporter assay (Luc) system (Qiagen) was chosen for monitoring HIF- and NFκB-Luc pathway activity in fibroblasts, as we previously described [46, 47]. Luciferase assay (E1501, Promega) was performed in luciferase reporter hTERT-BJ1 cells treated with atovaquone. Briefly, 1 × 10^4^ hTERT-BJ1 reporter cells were seeded in black-walled 96-well plates and then were treated with atovaquone (5μM and 10 μM) for 48 hours. Vehicle alone (DMSO) control cells were run in parallel. Six replicates were used for each condition. After 48 hours, luciferase assay was performed according to the manufacturer's instructions. Light signal was acquired for 2 minutes in photons/second in the Xenogen VivoVision IVIS Lumina (Caliper Life Sciences), and the results were analyzed using the Living Image 3.2 software (Caliper Life Sciences). Luminescence was normalized using SRB (total proteins), as a measure of cell viability.

### Statistical analysis

Statistical significance was measured using the analysis of variance (ANOVA) test or *t*-test. P ≤ 0.05 was considered significant and all statistical tests were two-sided.

### Kaplan-Meier (K-M) analyses

To perform K-M analysis, we used an open-access online survival analysis tool to interrogate publically available microarray data from up to 2,609 breast cancer patients. This allowed us to determine their prognostic value. Biased and outlier array data were excluded from the analysis. Hazard-ratios were calculated, at the best auto-selected cut-off, and p-values were calculated using the logrank test and plotted in R. K-M curves were generated online using the K-M-plotter (as high-resolution TIFF files), using univariate analysis: http://kmplot.com/analysis/index.php?p=service&cancer=breast. This allowed us to directly perform in silico validation of these mitochondrial biomarker candidates.

## SUPPLEMENTARY MATERIAL FIGURES


