# Supplementary material for: Repurposing atovaquone: Targeting mitochondrial complex III and OXPHOS to eradicate cancer stem cells
Source: Oncotarget. 2016 Apr 30;7(23):34084–99. doi: 10.18632/oncotarget.9122 (PMC5085139; doi:10.18632/oncotarget.9122)
Supplement: Supplementary file 1 [file oncotarget-07-34084-s001.pdf]

Repurposing atovaquone: Targeting mitochondrial complex III and OXPHOS to eradicate cancer stem cells

Supplementary Material: Figure S1

A

All Breast Cancers

RFS

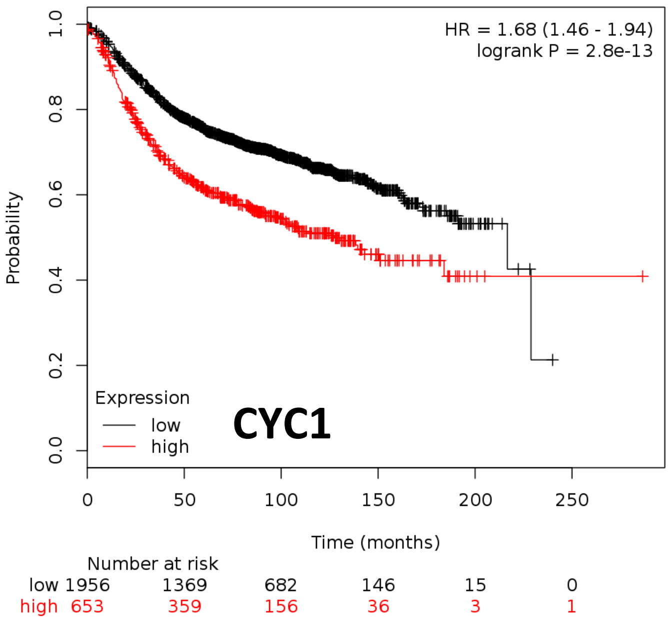

N=2,609

B

ER(-) Breast Cancers

RFS

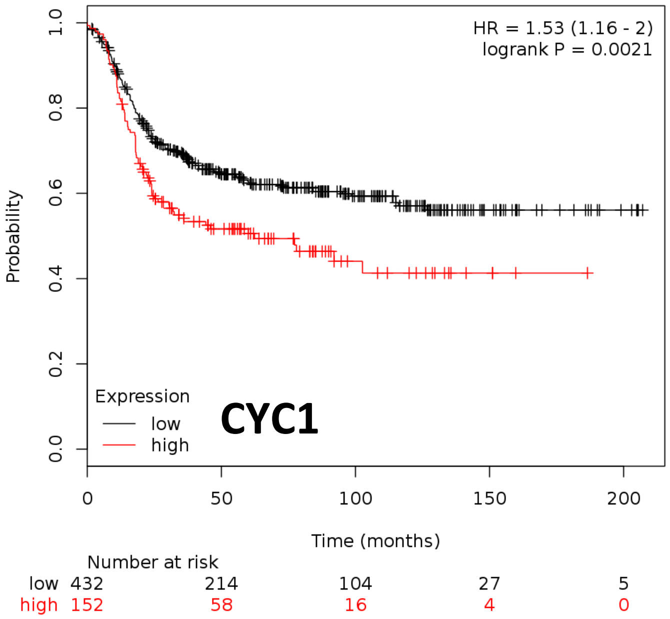

N=584

C

ER(-)/Basal/Breast Cancers

RFS

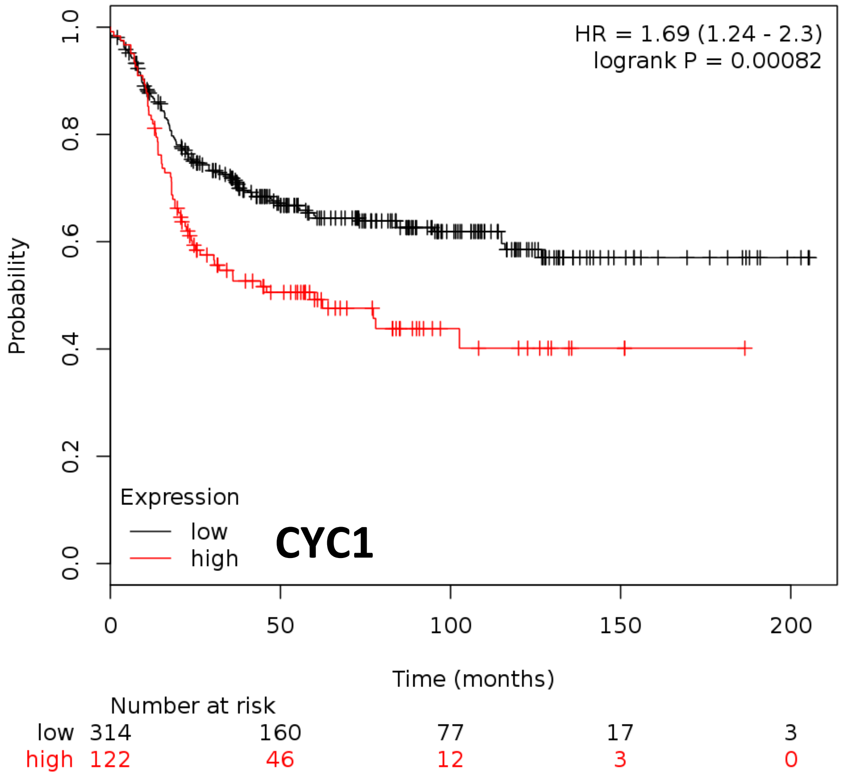

N=436

D

ER(+) Breast Cancers

RFS

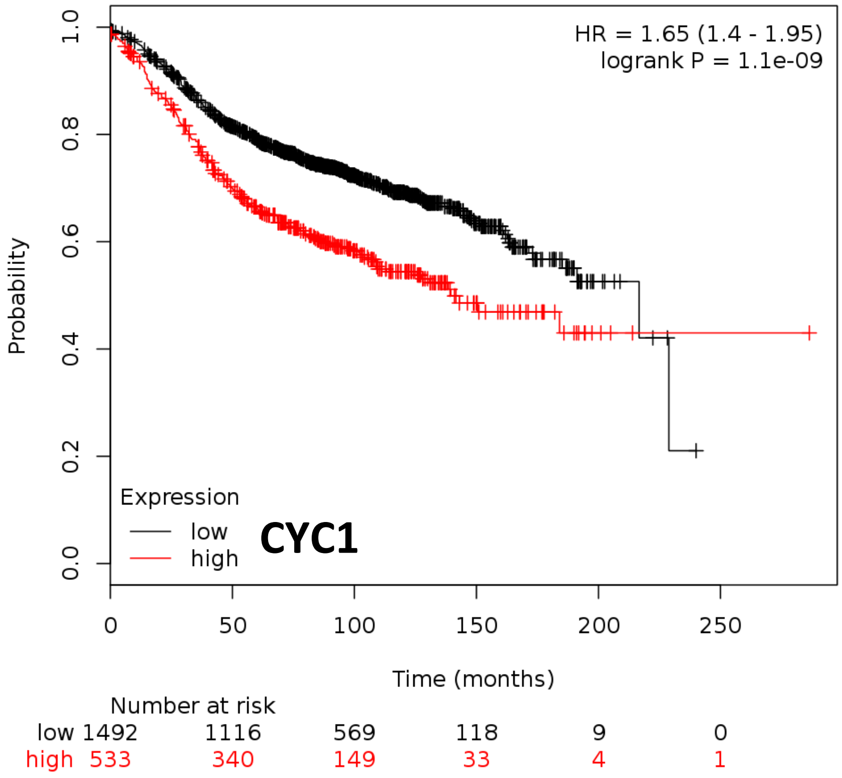

N=2,025

E

ER(+)/Endocrine Therapy

RFS

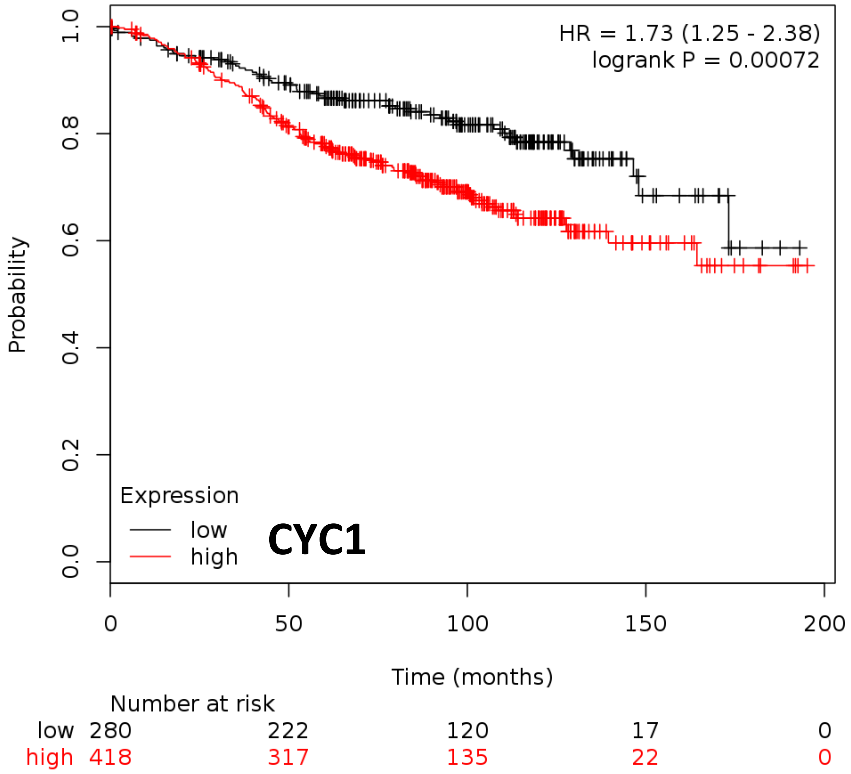

N=698

F

ER(+) Luminal A/LN(+)/ Endocrine Therapy

RFS

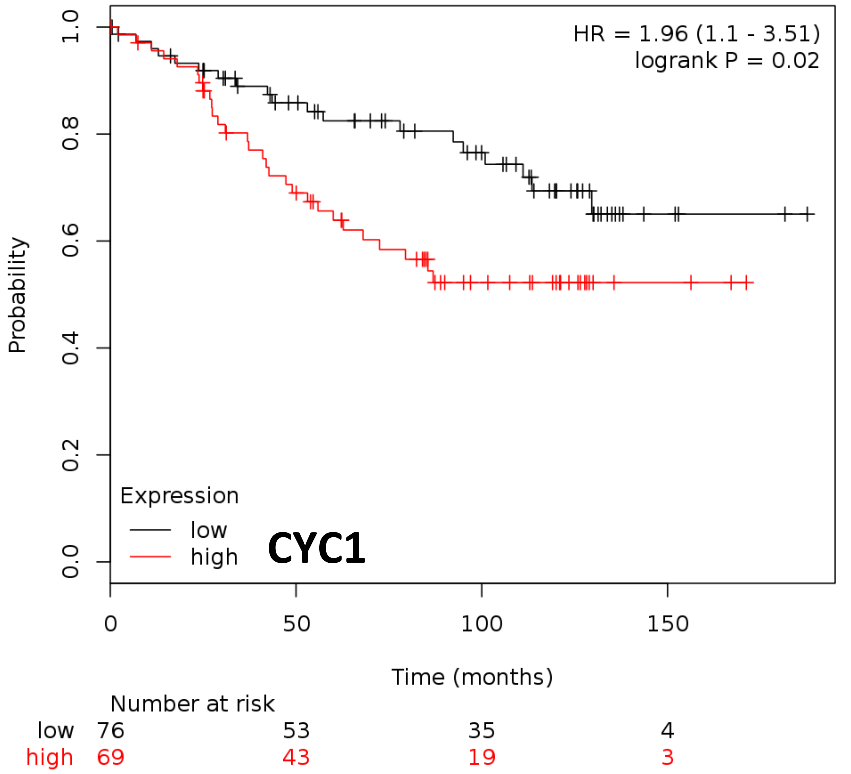

N=145

G

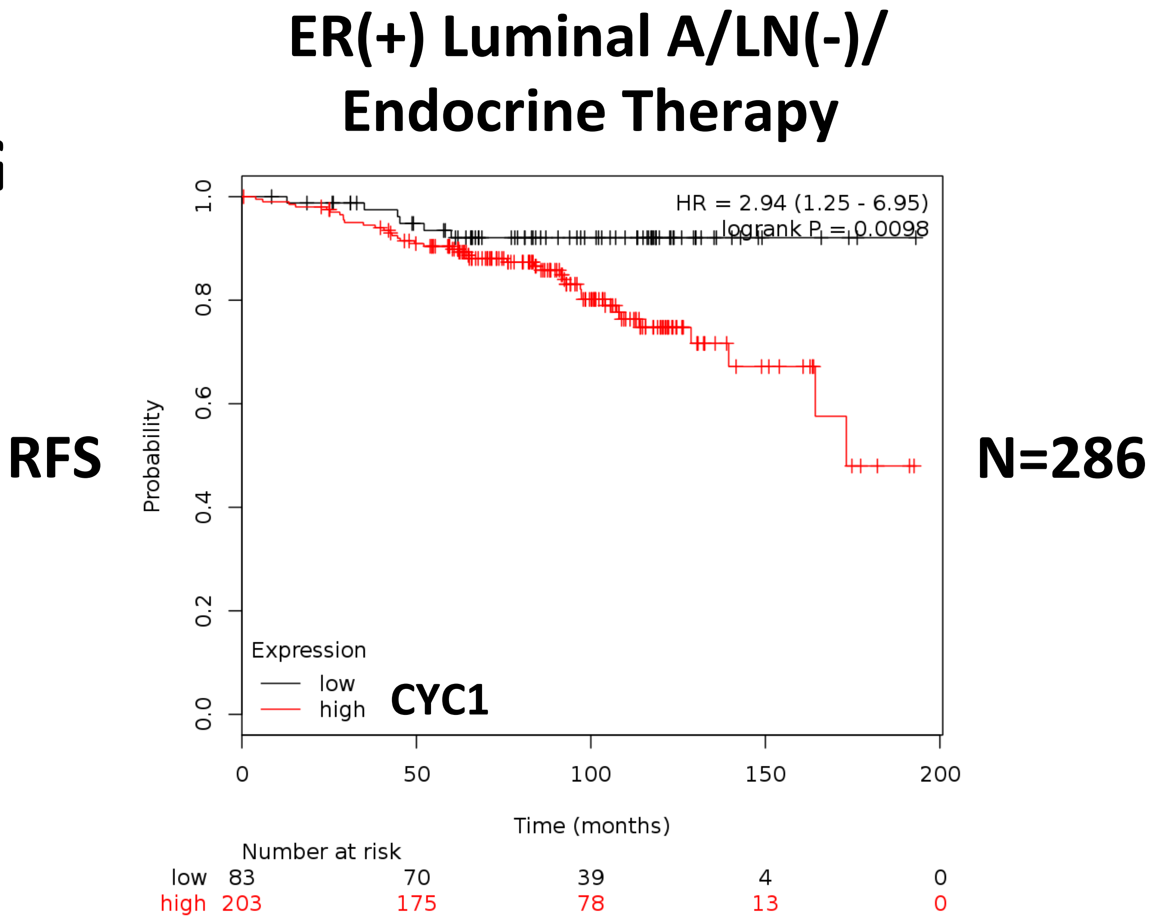
ER(+) Luminal A/LN(-)/
Endocrine Therapy
RFS
N=286
Probability
0.0
0.2
0.4
0.6
0.8
1.0
HR = 2.94 (1.25 - 6.95)
logrank P = 0.0098
Expression
low
high
CYC1
0
50
100
150
200
Time (months)
Number at risk
low 83 70 39 4 0
high 203 175 78 13 0

H

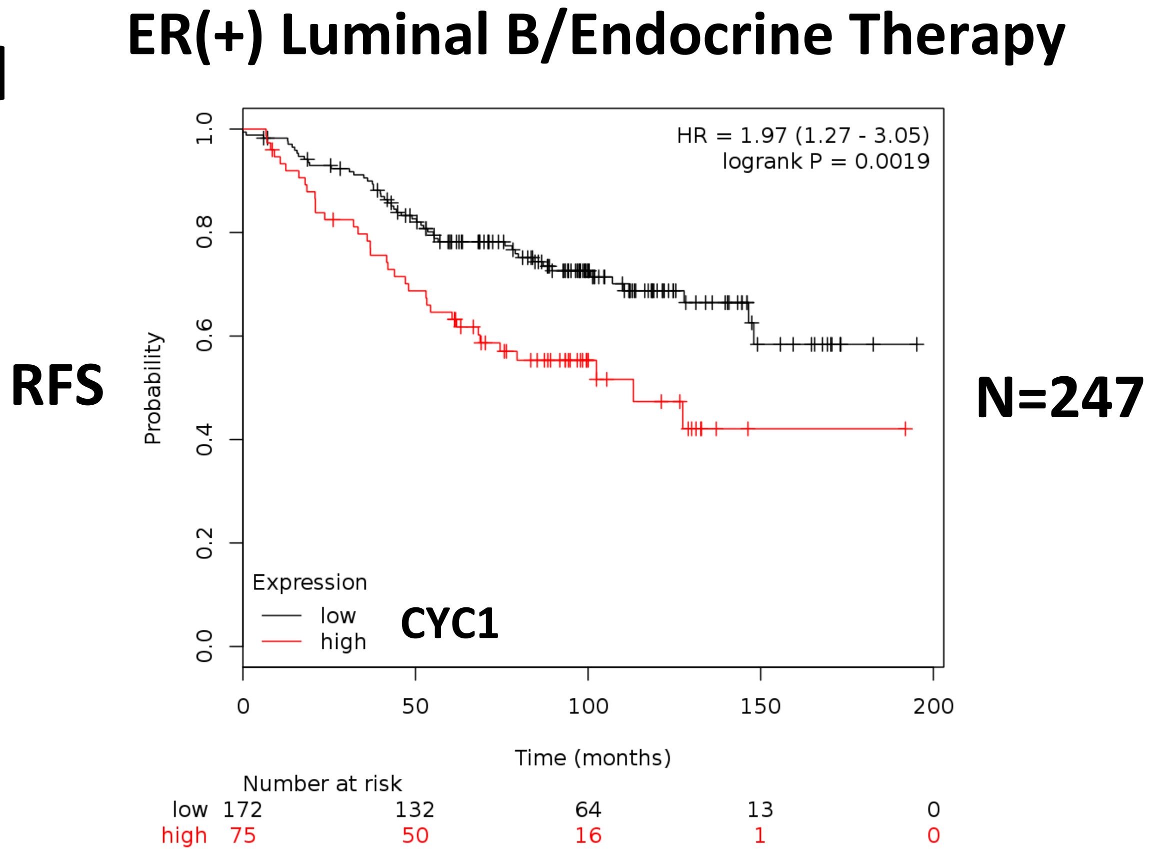
ER(+) Luminal B/Endocrine Therapy
RFS
N=247
Probability
0.0
0.2
0.4
0.6
0.8
1.0
HR = 1.97 (1.27 - 3.05)
logrank P = 0.0019
Expression
low
high
CYC1
0
50
100
150
200
Time (months)
Number at risk
low 172 132 64 13 0
high 75 50 16 1 0

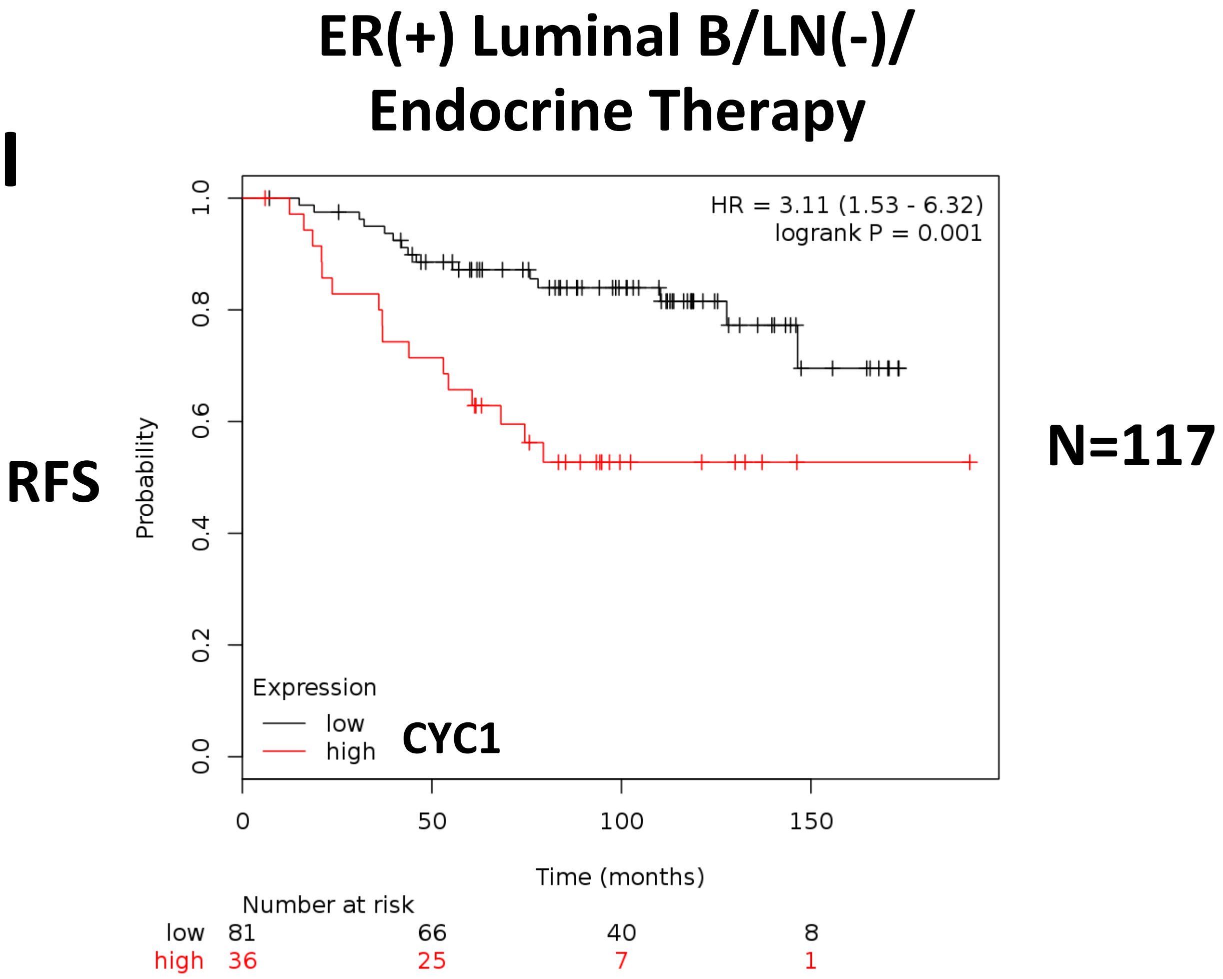

Supplementary Figure S1: K-M plots of RFS (Recurrence-free Survival) are shown for CYC1 expression in different sub- groups of breast cancer patients (Panesls A-I). These results are summarized in Table 1.

Repurposing atovaquone: Targeting mitochondrial complex III and OXPHOS to eradicate cancer stem cells

Supplementary Material: <u>Figure S2</u>

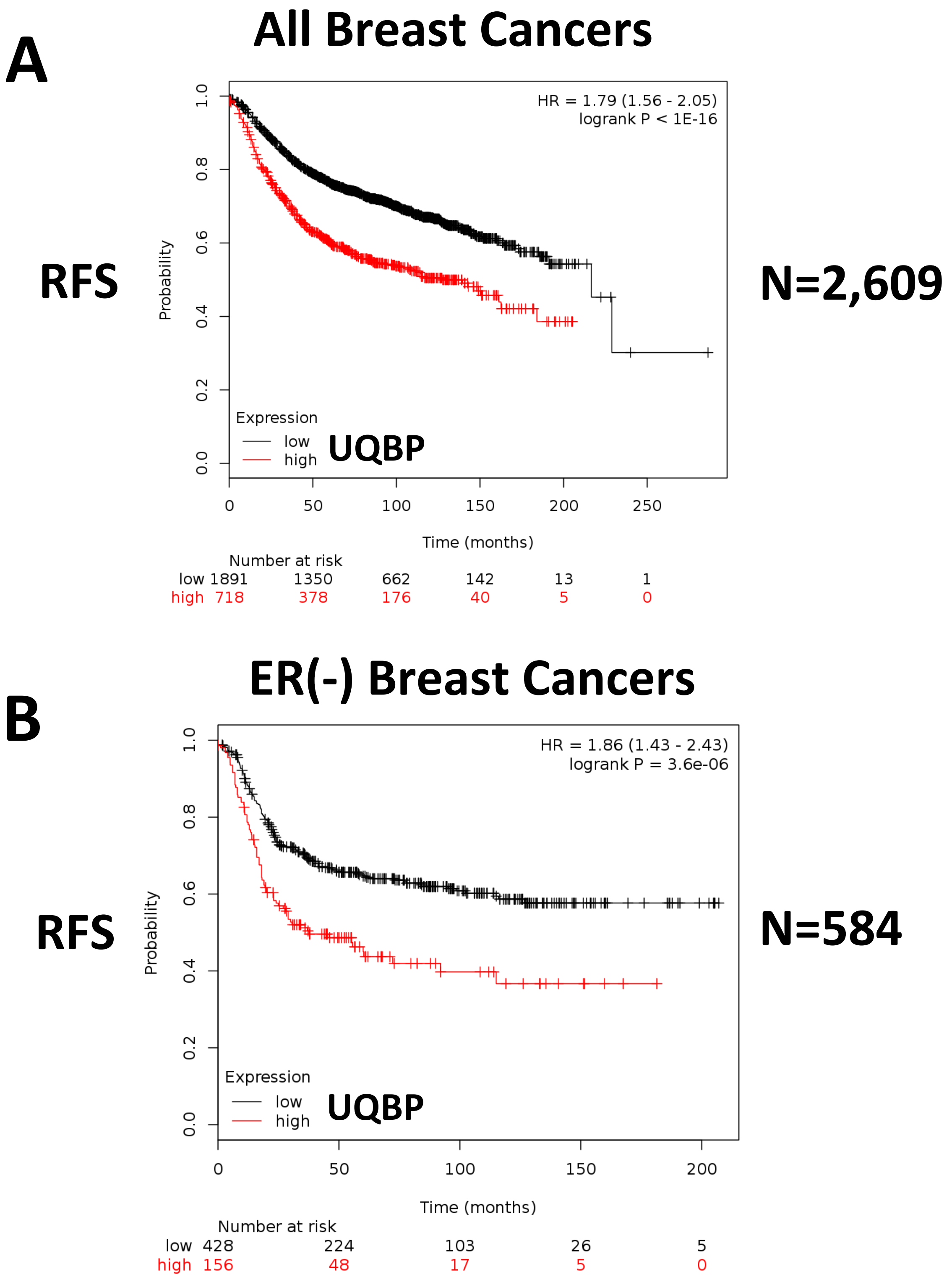

C

ER(-)/Basal/Breast Cancers

RFS

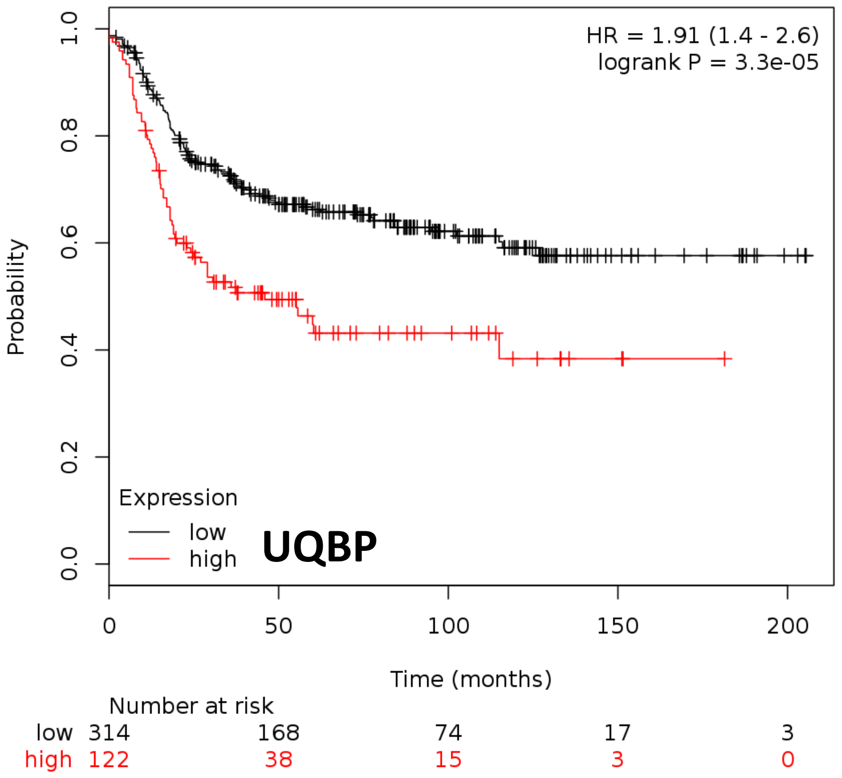

N=436

D

ER(+) Breast Cancers

RFS

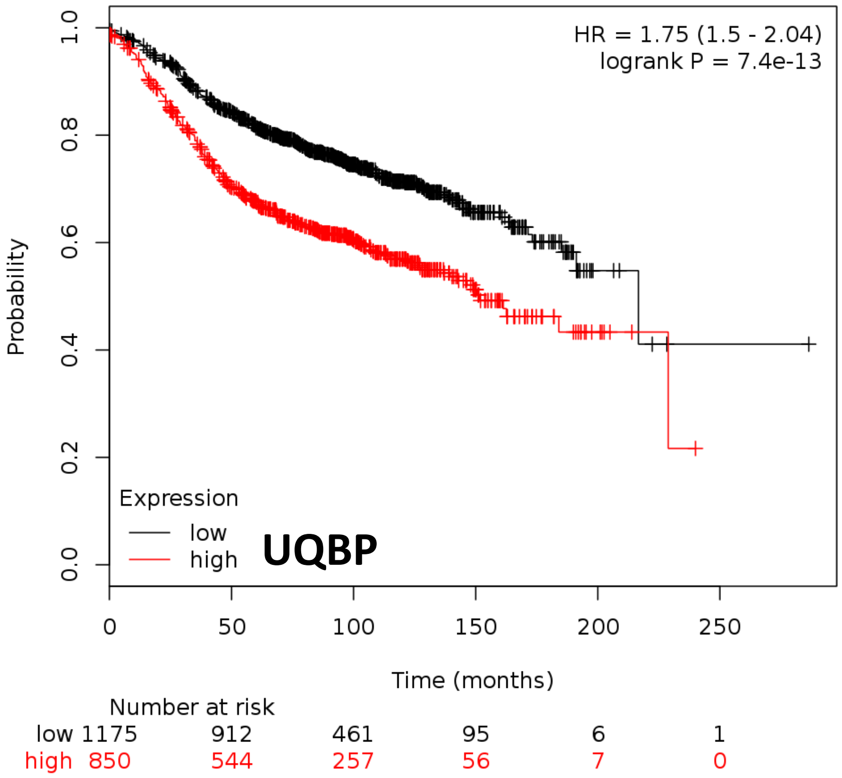

N=2,025

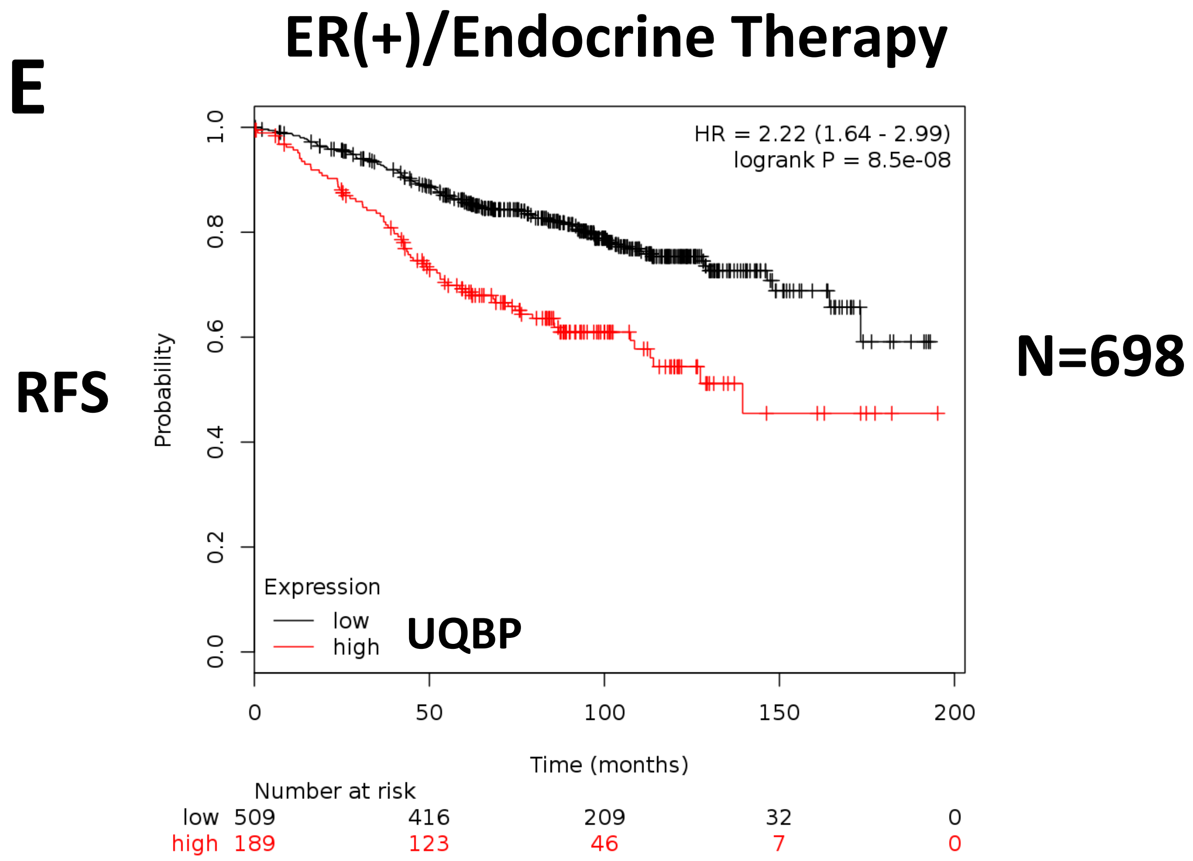
ER(+)/Endocrine Therapy
E
RFS
N=698
HR = 2.22 (1.64 - 2.99)
logrank P = 8.5e-08
Probability
Expression
low
high
UQBP
Time (months)
Number at risk
low 509 416 209 32 0
high 189 123 46 7 0

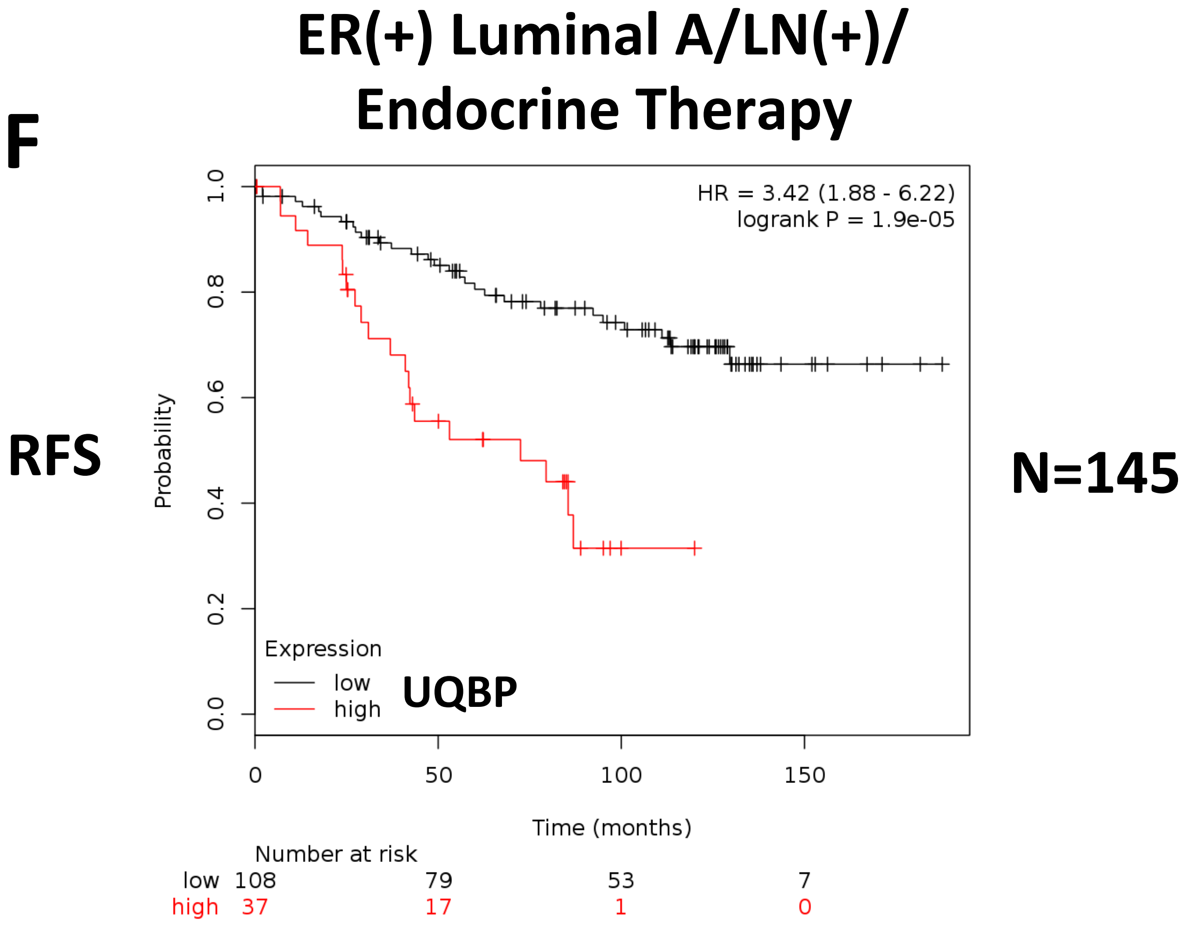
ER(+) Luminal A/LN(+)/
Endocrine Therapy
F
RFS
N=145
HR = 3.42 (1.88 - 6.22)
logrank P = 1.9e-05
Probability
Expression
low
high
UQBP
Time (months)
Number at risk
low 108 79 53 7
high 37 17 1 0

G

ER(+) Luminal A/LN(-)/ Endocrine Therapy

RFS

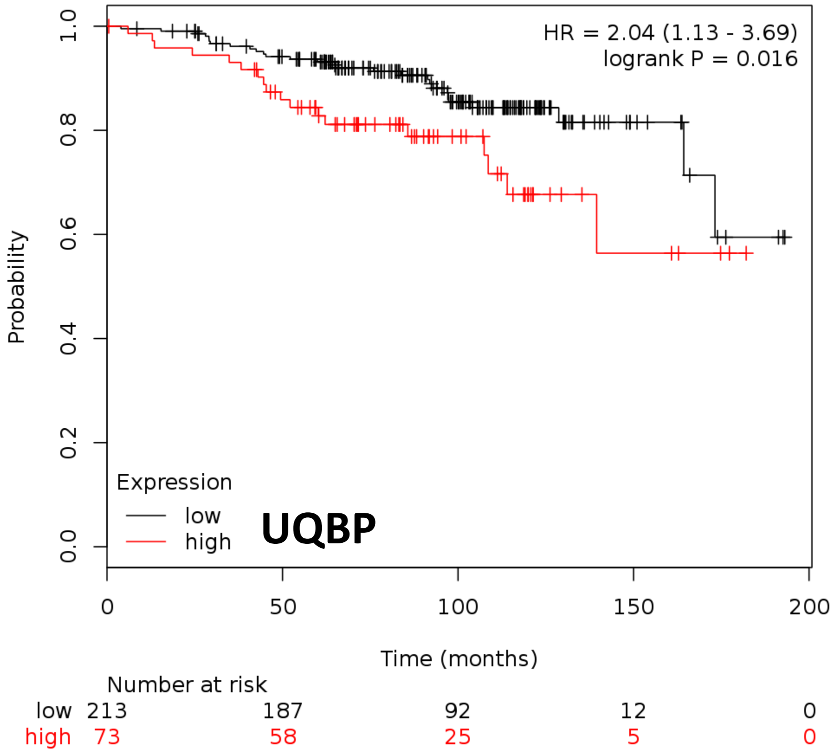

N=286

H

ER(+) Luminal B/Endocrine Therapy

RFS

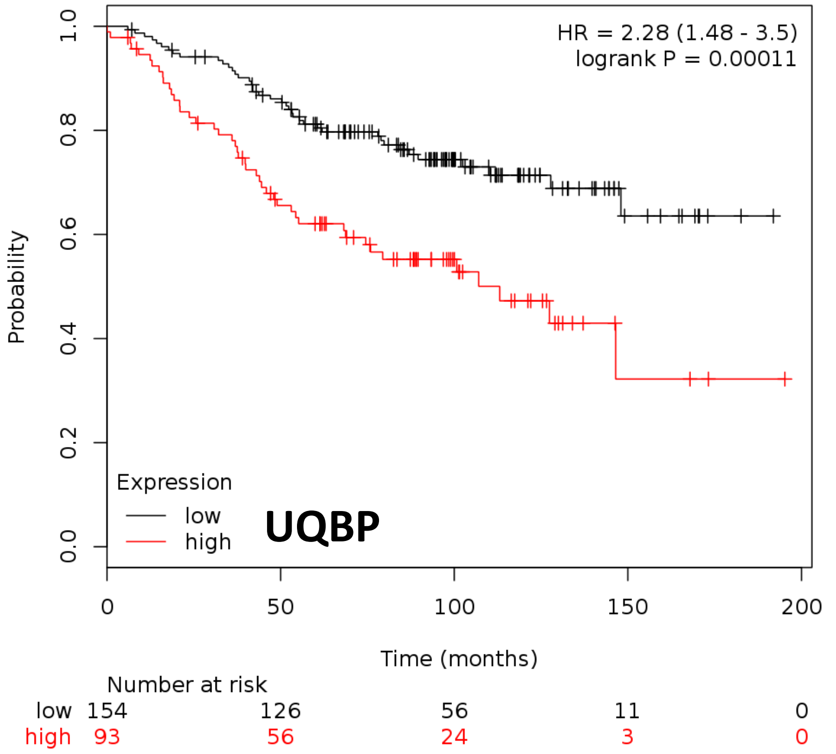

N=247

ER(+) Luminal B/LN(-)/ Endocrine Therapy

I

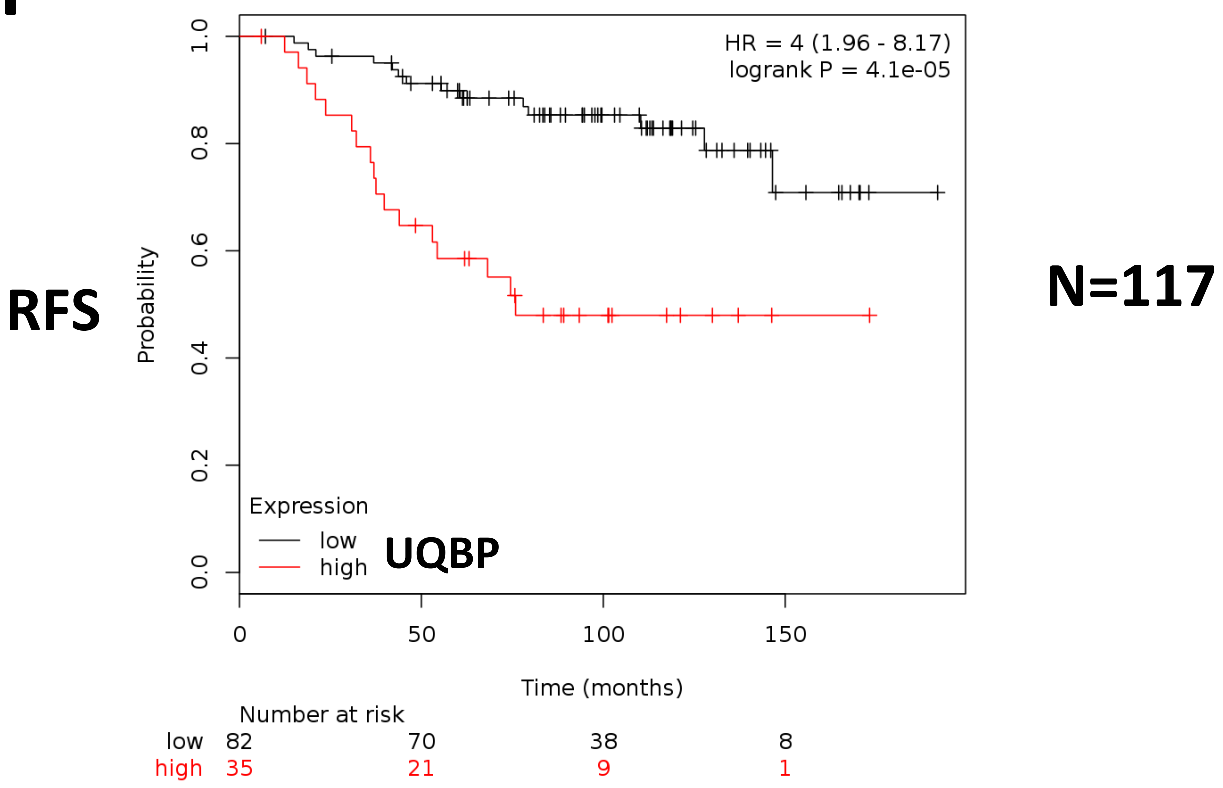

Supplementary Figure S2: K-M plots of RFS (Recurrence-free Survival) are shown for UQBP expression in different sub- groups of breast cancer patients (Panesls A-I). These results are summarized in Table 2.
